# E-Learning Satisfaction, Stress, Quality of Life, and Coping: A Cross-Sectional Study in Italian University Students a Year after the COVID-19 Pandemic Began

**DOI:** 10.3390/ijerph19138214

**Published:** 2022-07-05

**Authors:** Vincenza Cofini, Enrico Perilli, Annalucia Moretti, Valeria Bianchini, Matteo Perazzini, Mario Muselli, Sabrina Lanzi, Loreta Tobia, Leila Fabiani, Stefano Necozione

**Affiliations:** Department of Life, Health and Environmental Sciences, University of L′Aquila, 67100 L′Aquila, Italy; vincenza.cofini@univaq.it (V.C.); enrico.perilli@univaq.it (E.P.); valeria.bianchini@univaq.it (V.B.); matteo.perazzini@student.univaq.it (M.P.); mario.muselli@graduate.univaq.it (M.M.); sabrina.lanzi@student.univaq.it (S.L.); loreta.tobia@univaq.it (L.T.); leila.fabiani@univaq.it (L.F.); stefano.necozione@univaq.it (S.N.)

**Keywords:** medical education, e-learning, distance learning, COVID-19 pandemic, training satisfaction, college students, COVID-19, students

## Abstract

(1) Background: The objective was to investigate e-learning satisfaction in a sample of university students by evaluating quality of life, stress sociality, and coping. (2) Methods: This was an online survey involved 471 students attending the University of L′Aquila from June to July 2021. The primary goal was estimating the e-learning satisfaction as measured by the E-learning Satisfaction Scale, while the secondary outcomes were studying its relationship with demographic factors, the perception of quality of life, sociality, stress, and coping strategies using a hierarchical regression model. (3) Results: A total of 136 participants were males (29%); the mean age was 25 years. The results revealed that the satisfaction score was 30.6, and the mean stress level was 19.4. Students suffered psychologically and physically for 14 days a month. The sociality score was 36. With respect to coping strategies, students reported higher scores for “Acceptance” (6.0), “Active coping” (6.2), and “Planning” (6.4). E-learning satisfaction was significantly related to age and course attendance. It was positively associated with the social presence score and coping strategies such as self-blame and religion, while it was inversely related to stress and unhealthy days. (4) Conclusions: The students revealed a positive propensity to use e-learning despite the end of quarantine. Sociality, stress, quality of life, and coping seemed to play an important role in student′s e-learning satisfaction.

## 1. Introduction

The pandemic event due to COVID-19 and the consequent lockdown in many countries of the world caused the closure of schools and universities in 2020, and in many countries, governments activated measures to maintain the continuity of learning using distance learning [1].

Since the end of February 2020, Italy has been one of the countries most affected by SARS-CoV-2, and similarly to other sectors, the educational sector has been affected by the pandemic situation. On 4 March 2020, the Government suspended all educational activities, then on 8 March 2020, a national quarantine was announced, restricting population mobility except for necessity, work, and health circumstances. In this situation, distance learning emerged as a new teaching method never adopted before, maintaining medical education continuity. The national lockdown ended on 4 May 2020, but distance teaching was maintained. In an emergency, online learning may be a suitable alternative to traditional learning to maintain the continuity of educational activities. However, clinical consequences and the continuous spread of the epidemic, the forced social isolation measures, and delays in starting schools and universities across the country were expected to influence college students′ mental health with long-term psychological pressure [2]. Moreover, the persistence of the state of emergency and uncertainties about the future have made distance learning an increasingly important everyday tool, so it is necessary to question its usefulness in delivering knowledge. The situation caused by the coronavirus pandemic forced the adoption of new distance-learning methods that had never been routinely engaged on such a large scale [3]. This forced students to cope with new methods, technologies, and changing environments, and to protect themselves and others from the spread of COVID-19.

Fortunately, technological developments made it possible to continue teaching via e-learning, instead of there being a total interruption of it. In fact, the evolution of technology and accessibility to the Internet has increased interest in the educational use of technological devices over the past 20 years [4], although some researchers argue that the didactic performance resulting from online learning is questionable because it results in the absence of a direct and face-to-face relationship between teacher and learner [5].

In addition, properly planned online learning experiences must be differentiated from courses presented online as a response to the state of emergency due to the COVID-19 pandemic.

Adequately e-learning planning has been shown to influence students′ satisfaction and the perceived usefulness of e-learning. Particularly, students′ satisfaction with e-learning depends on timely and effective feedback from teachers, high-quality and regularly updated course content, as well as user-friendly features such as receiving appropriate technical support and the presence of features that promote the completion of learning-related tasks efficiently and successfully [6].

Learning satisfaction represents learners′ feelings and attitudes toward the learning process, or the perceived level of fulfillment attached to one′s desire to learn caused by the learning experiences [7].

According to Weerasinghe and Elliott [8,9], student satisfaction is a short-term attitude based on a review of the educational experience, services, and facilities provided to students. One of the most essential criteria examined when evaluating achievement in the deployment of a system in the e-learning setting is student happiness. Some characteristics that contribute to student happiness in the e-learning environment were discovered in previous studies. In their review study, Nortving, Peterson, and Balle [10] found that many factors were significantly related to student satisfaction: instructor presence in online settings; interactions among students, teachers, and content; and planned connections between online and offline activities and campus-related and practice-related activities.

Furthermore, the use of e-learning evidenced the importance of also implementing teachers′ technological and digital competencies, which have an important role during online lessons beyond the pedagogical skills [11].

The distance learning provided in many countries, including Italy, during the state of emergency was a response to the lockdown and not an adequately planned distance learning [12].

Various studies carried out during lockdown that focused on undergraduate students′ perception of the learning environment showed positive perception of distance education and negative experiences of psychological distress such as anxiety, with anxiety also being higher among females [13]. The COVID-19 pandemic had a negative effect on the psychological well-being of university students worldwide, as reported by several studies [14,15]. In addition, a study carried out in Sudan in 2021 on a group of 252 undergraduate pharmacy students showed a high prevalence of academic stress among participants (80%), with memory difficulties and concern for exams during the COVID-19 pandemic [16].

In addition, online learning has had a negative impact on the private and academic life of students. Due to conflicts in responsibilities with everyday household activities and family or personal commitments at home, environmental influences at home may be unavoidable. Furthermore, students′ families or household members may not have recognized the necessity for privacy and quiet when engaging in online education. Students may also have difficulties as a result of their scholastic obligations as students colliding with their roles as family members in performing allocated family tasks and duties during the epidemic [17].

Other studies have shown that overall e-learning quality has a significant positive impact on student satisfaction and intentions to continue usage of e-learning platforms [18,19,20].

Additionally, another study conducted in China on a sample of 1504 university students showed a positive relation between interaction and online learning satisfaction, interaction and academic self-efficacy, academic self-efficacy and student engagement, and student engagement and online learning satisfaction [21].

Another study showed that there was a direct relationship between individual self-efficacy and satisfaction with virtual learning [22].

Students′ coping strategies used during the pandemic might have influenced their adaptation and propensity to engage in e-learning.

Coping refers to specific cognitive and behavioral strategies for mastering, reducing, or tolerating internal and external demands to stressful situations [23]. These coping strategies can be positive or negative [24]. Positive coping is an active coping style that focuses on taking constructive actions and changing the stressful situation, and it is typically associated with problem-solving behavior and effective emotion regulation [25]. In contrast, negative coping is a passive style focusing on negative appraisals and emotional expression, escape from stressful situations, and social isolation [25].

To cope with academic stress in the pandemic situation, students put in place several strategies. Some engaged in many creative activities and began learning new hobbies; others started a voluntary internship (working from home) with companies; still others engaged in online courses. Other students instead were seduced by the Internet and spent much of their time on social media and on e-connecting with others. Students were attempting to cope with the current epidemic condition by employing their emotional intelligence and deflecting boredom and sad thoughts [26].

Additionally, interpersonal relationships in a university community may be perceived differently in face-to-face lessons or virtual lessons; thus, e-learning satisfaction may be different.

Social presence, or the feeling of being with a “real” person, is a fundamental component of the interactions that occur in virtual reality. One of the main points of interest of virtual reality is the level of social presence it offers compared to other forms of communication mediated by technology. Social presence is related to the subjective experience of having access to the thoughts and emotions of a real person [27,28].

Social presence is supported by the use of computer-mediated communication (CMC) tools and electronic platforms for online group learning (OGL). Social presence influences how social interaction in OGL groups takes place online and, in turn, affects learning outcomes [29,30]. Today, we observe how technology has evolved further and goes beyond simple text CMC; due to the recent COVID-19 pandemic, there has been a growth in the use of real-time video communication applications, including Zoom, Teams, Meeting [28], and Discord [31].

However, despite its importance, the precise definition of social presence has been debated, as there is currently a wide range of definitions of social presence and measures to assess it [32]. Charlotte N. Gunawardena et al. found that social presence is a predictor of a student′s satisfaction during computer conferences [33].

We hypothesized that satisfaction with e-learning that was delivered suddenly and forcibly could be linked to the perception of one′s well-being as severely tested by the pandemic, and with the ability to cope with the new academic and social situation.

This study was conducted to understand and analyze the satisfaction with distance education experienced by a sample of Italian university students independent of the type of technology used and the technical skills of students and teachers. The COVID-19 pandemic caused a very high death toll in Italy and, in our university, although the technology was largely used, the learning activities were traditional, and there was no time to do otherwise. Concern for academic careers in the new context and concern about not getting sick had a strong impact on the well-being of young people.

According to our knowledge, no study, at least in Italy, has investigated the fact that the perception of quality of life, stress, social presence, and coping could be predictors of the satisfaction with distance education during the epochal event of the pandemic, in which the change from traditional face-to-face teaching to remote teaching was organized quickly with the sole purpose of not interrupting the students′ careers.

The primary goal of the present study was estimating e-learning satisfaction. Therefore, we investigated the stress perceived by the students, the perception of the quality of life, and social presence. We also attempted to analyze the coping strategies of students faced with the COVID-19 emergency.

The secondary objective was to investigate whether sociodemographic factors, psychological distress (stress, quality of life), social presence, and coping strategies were related to e-learning satisfaction.

## 2. Materials and Methods

This study was designed as an observational cross-sectional study on a sample of students attending medical courses, health science courses, and other courses at the University of L′Aquila in Abruzzo, a region of Southern Italy. The study was presented by six teachers from three different departments during two webinars, and it was a part of a larger research project was authorized by the internal review board (IRB) of the university. Participation was voluntary. All students were invited to participate in a Web-based online survey by filling out a questionnaire after giving informed consent by the (specific) apposite flag.

The questionnaire was composed of five validated scales: “E-learning Satisfaction Scale”, “Health-related Quality of Life ((HRQoL)”, “Social Presence Scale”, “General Health Questionnaire (GH-12)”, and “Brief COPE (BC) scale” (Supplementary Materials).

### 2.1. E-Learning Satisfaction Scale

To evaluate distance education, we used an adapted version of the E-learning Satisfaction Scale. The original questionnaire consisted of nine items scored on a Likert scale of 1–5. A score of 1 = strongly disagree, 2 = disagree, 3 = uncertain, 4 = agree, and 5 = strongly agree. [33,34]. The maximum possible score was 45, and a high level indicated high satisfaction. Reliability was reported as 0.87 using Cronbach′s alpha, sufficient according to Carmines and Zeller [35]. Validity data are not presented. In our study, we used 8 items from the “Satisfaction Scale”, and we added two items “Professor was available and helpful in facilitating the use of the platform” and “I had difficulty concentrating”; the Cronbach′s alpha that resulted was 0.89.

### 2.2. Health-Related Quality of Life (HRQoL)

The Health-related Quality of Life (HRQoL), developed by the Centers for Disease Control and Prevention and used in the Italian Epidemiological Surveillance System in adult people (PASSI), was used to assess the “Summary Index of Unhealthy Days”, which is a validated index of self-reported mental and physical health that allows researchers to track health trends over time and identify groups of people who may require special treatment [36,37,38,39,40].

The HRQoL is based on the answers to four core questions [41]:

I. How is your health in general? (Excellent, Very good, Good, Fair, or Poor).
II. Now, thinking about your physical health, which includes physical illness and injury, how many days during the past 30 days was your physical health not good?
III. Now, thinking about your mental health, which includes stress, depression, and problems with emotions, how many days during the past 30 days was your mental health not good?
IV. Now, think about your usual activities. During the past 30 days, approximately how many days did poor physical or mental health keep you from doing your usual activities, such as self-care, work, or recreation?

The “Summary Index of Unhealthy Days,” with a maximum of 30 days, was calculated using the two HRQOL questions on physical and mental health.

### 2.3. Social Presence Scale

To measure the level of social presence, we used the “Social Presence Scale” (SPRES), introduced by Gunawardena and Zittle (1997). This questionnaire [33] consists of 12 items that embody the concept of “immediacy” as defined in Short et al. [42]. To simplify the administration of the questionnaire, we omitted the last two questions because they were not applicable to the context of a university lecture. A Likert scale was used, with scores ranging from 1–5. A score of 1 = strongly disagree, 2 = disagree, 3 = uncertain, 4 = agree, and 5 = strongly agree. The maximum possible score was 70 [43].

A slight modification to the wording of the scale was made as appropriate for a Web-based epidemiology course.

### 2.4. General Health Questionnaire (GH-12)

The General Health Questionnaire (GH-12) consists of a 12-item scale used to assess perceived psychological distress: each item assesses the severity of a mental problem over the past few weeks using a 4-point scale with a Likert scoring method (from 0 to 3); the possible answers are “less than usual”(score = 0), “no more than usual” (score = 1), “rather more than usual”(score = 2), or “much more than usual” (score = 3). The 12 items evaluated are: able to concentrate, lost sleep over worry, play useful part in things, capable of making decisions, constantly under strain, could not overcome difficulties, enjoy day-to-day activities, face up to problems, feeling unhappy and depressed, losing confidence in self, thinking of self as worthless, and reasonably happy. A GH12 score ≤ 15 was used to indicate an average stress level, a 15–20 score indicated a moderate level of stress, and a score ≥ 20 indicated more intense psychological distress [44].

### 2.5. Brief COPE Scale

The Brief COPE (BC) is the abridged version of the COPE inventory. It includes 28 items that assess 14 thematically distinct coping reactions: self-distraction, denial, active coping, substance use, use of emotional support, use of instrumental support, behavioral disengagement, venting, positive framing, planning, humor, acceptance, religion, and self-blame [45,46,47]. Some of these responses are well-known to be adaptive, whereas others are well-known to be harmful. As a result, the Brief COPE allows researchers to swiftly measure potentially relevant coping reactions. Response options range from 0 (I have not been doing this at A/I) to 3 (I have been doing this a lot).

The questionnaire also included items on sociodemographic information: gender, age, residence, and type of course being attended. Data were collected from June 2021 to July 2021, a year after the beginning of the COVID-19 pandemic in Italy.

### 2.6. Outcomes

The primary outcome was to evaluate e-learning satisfaction. The secondary outcome was studying its relationship with sociodemographic factors, the perception of quality of life, social presence, stress, and coping strategies.

### 2.7. Sample

The sample size was calculated according to the total number of students at the University of L′Aquila for the academic year 2020/2021 (18769). Using the Raosoft software′s sample-size calculator (http://www.raosoft.com/samplesize.html, accessed on 6 June 2021) with a confidence level of 95%, a margin of error of 5%, and a response distribution of 50%, the lower limit of participants who should have been included was 377 students. Snowball sampling was used to select the study participants.

### 2.8. Statistical Analysis

All variables were analyzed reporting proportions for categorical data and means and standard deviations (SDs) for continuous data. Continuous variables were tested for normality with the Shapiro test and SD test for equality of standard deviations.

Before constructing the final multivariable model to analyze the data collected, we ran a univariate analysis to explore the relationships between the outcome (e-learning satisfaction) and each independent variable separately, using statistic tests to compare means between groups for categorical variables and the Spearman correlation coefficient for quantitative variables. Therefore, a t-sample *t* test was used to compare the means of the e-learning satisfaction score between males and females, while a one-way ANOVA model was run to evaluate the differences in e-learning satisfaction between groups with respect to the residence (South, Central, or Northern Italy) and course attended (medicine/health Science/other courses); multiple comparisons were adjusted with the Bonferroni method.

The Spearman correlation, as introduced above, was run to investigate the relation between e-learning satisfaction and the following variables: age, social presence (SPRES score), stress (GHQ-12), health-related quality of life (H-RQoL: Summary Index of Unhealthy Days), and coping strategies (brief-coping subscale scores).

A hierarchical multiple regression analysis was conducted to assess the association of the outcome (e-learning satisfaction) with sociodemographics (gender, age, attending course, residence), stress, health-related quality of life (H-RQoL: Summary Index of Unhealthy Days), social presence, and coping variables.

All variables investigated were entered into the hierarchical regression analysis using a variance-covariance matrix of the estimators (vce) set to robust.

The variables were entered in the following order: demographics to control for confounding of variables that may be associated with e-learning satisfaction (Block 1); followed by stress, health-related quality of life (H-RQoL: Summary Index of Unhealthy Days), and social presence (Block 2), which has been evidenced in other research to be associated with e-learning satisfaction [20,33,48] brief-coping subscales (Block 3), which were explored as new elements in this article. For the model regression coefficients, 95% CI, F statistics, R2, and change in F and R2 are reported.

To evaluate the multicollinearity, a variance inflation factor (VIF) was used; to investigate the residuals′ distribution, a Q–Q plot of residuals was plotted and tested for normality. The confidence level was set to 5%, and all analyses were performed using the Stata 14 software package.

## 3. Results

Four hundred forty-seven students participated in the study. They were 25 years old on average and were prevalently females (335/471), with 136 males (29%). Most of the students reported residing in Southern Italy, as expected (295/471). A total of 47% were medical students, 131 were in health science courses, and 117 were in different courses classified as “Others” (Table 1).

### 3.1. E-Learning Satisfaction

The students reported a score for e-learning satisfaction of 30.6 (SD:7.1) with a median value of 30, indicating a good level of satisfaction, with scores ranging from 13 to 47. Table 2 reports the mean and SD for all items of the E-learning Satisfaction Scale used.

### 3.2. Stress, Health-Related Quality of Life, Social Presence, and Coping Strategies

Table 3 reports a descriptive analysis of all factors related to the well-being and coping investigated in the sample. The mean level of perceived stress was 19, which was classifiable as a moderate stress level, as reported in the Section 2.

The “Health-related Quality of Life” measures showed that students suffered in their physical and mental health. They in fact perceived, on average, 14 unhealthy days a month, as indicated by the Summary Index of Unhealthy Days; the percentage of students who perceived more than 14 unhealthy days was 47% (data not reported in Table 3).

With respect to “Social Presence” as reported by the SPRES score, students reported, on average, a level of 36.

Students reported higher scores for the following coping strategies: “Acceptance” (6.0), “Active coping” (6.2), and “Planning” (6.4), while they indicated a lower score for “Substance use”.

### 3.3. Univariate Analysis

When exploring the relationship between the e-learning satisfaction score and each independent variable investigated, the univariate analysis showed that e-learning satisfaction was not different between males and females (t = −0.234; *p* = 0.816) and there were no differences among residence groups (F = 0.69; *p* = 0.500), but it was significantly related to course attendance (F = 5.35; *p* = 0.005), as significant differences were found between students in medicine and other courses (*p* Bonferroni < 0.05).

As reported in Table 4, the Spearman correlation analysis showed that the satisfaction increased with increasing age (rho = 0.3023; *p* = 0.0000). It was lower in those who perceived higher levels of stress and in students who lived a greater number of days in poor physical and mental health in terms of unhealthy days; in fact, the e-learning satisfaction score was inversely correlated with stress and unhealthy days (rho = −0.4350, *p* = 0.0000; and rho = −0.1830, *p* = 0.0000, respectively). Students with a high level of social presence had a high level of satisfaction with e-learning, and the correlation was strongly positive (rho = 0.7911, *p* = 0.0000). We found a positive correlation between the E-learning Satisfaction Scale and some subscales of brief scope. Most e-learning-satisfied students had higher scores in the subscales of active coping (rho = 0.1664, *p* = 0.0003), positive reframing (rho = 0.7911, *p* = 0.0044), planning (rho = 0.0927, *p* = 0.0043), and religion (rho = 0.2853; *p* = 0.0000). Instead, a negative correlation was found between e-learning satisfaction and substance use (rho = −0.1471; *p* = 0.0014) and behavioral disengagement (rho = −0.1092; *p* = 0.0178).

### 3.4. Multivariable Analysis

We ran the hierarchical regression model following three steps according to the three blocks indicated in Section 2, resulting in the three models reported in Table 5. In the first model (Table 5), age was the only variable to make a significant contribution to the model, with 11.2% of the variance in the e-learning satisfaction score explained. Sociodemographic factors, such as gender, course attendance (medicine/health sciences/others), and residence (Southern, Central, Northern Italy) were not factors associated significantly with e-learning satisfaction. In Model II, the sociodemographic variables were retained and stress, health-related quality of life, and social presence were added. Stress was negatively associated with e-learning satisfaction, while social presence was positively associated with it, explaining 66.7% of the variance. In Model III, 5 of the 12 variables made a significant contribution to the e-learning satisfaction scores.

The final model explained 68.6% of the variance in the e-learning satisfaction scores. E-learning satisfaction was positively associated with older age (beta = 0.123; *p* < 0.001), being a medical student (beta = 1.120; *p* < 0.05), social presence (beta = 0.601; *p* < 0.001), and the following coping strategies: religion (beta = 0.303; *p* < 0.05) and self-blame (beta = 0.435; *p* < 0.05); however, it was negatively associated with stress (beta = −0.121; *p* < 0.001).

## 4. Discussion

This observational study aimed to investigate e-learning satisfaction in a sample of Italian university students a year after the COVID-19 pandemic began in Italy.

According to our knowledge, this is the only study in Italy that attempted to study the relationship between the satisfaction of “forced” distance learning and aspects related to stress, the perception of physical and mental health, social presence, and the ability to cope with the new academic situation while at the same time limiting social life. Our results revealed that the investigated sample reported a good level of e-learning satisfaction, with a score of 30.6 on average (SD:7.1) during the COVID-19 pandemic.

In our sample, e-learning satisfaction was not related to gender or residence, but it was correlated with course attendance: medical students, compared to other courses evaluated, reported high levels of e-learning satisfaction. This finding was confirmed by research [49,50] that showed how medical students prefer e-learning over traditional methods. We assumed that medical students were more satisfied with the use of e-learning, as it allows them to link theoretical study to practical experiences. For example, we highlighted the usefulness of video clips for training on specific medical maneuvers, permitting the student to review and revise the clip [51]. Thanks to e-learning, teachers and students can interact even if they do not share the same physical space. In this way, e-learning can offer a solution to the problem of crowded classrooms, which can be particularly beneficial in first-year courses [52]. Another possible explanation for a higher level of satisfaction in medical students could be their computer and information technology proficiency; previous research reported that approximately 90% reported that they had good, very good, or proficient skill levels [53]. Furthermore, approximately 93% of the students reported that they owned a smartphone, while 75% had personal computers with economic benefits compared to other college students [53]. Furthermore, medical students, compared to other students in different courses, were more satisfied with e-learning, since they were more able to use adaptive coping strategies, as also supported in a recent study by Ruyue S. et al. [54].

The present study showed that satisfaction with e-learning increased with age. This finding was supported by some research [55], but contradicted by others [56] that found no significant relationship between student age and e-learning satisfaction. There may be various reasons for our findings, and the disparity between our findings and those of earlier research could be related to changes in the sample size, target demographics, or study scheduling [56]. Several reasons for our findings may exist, and this discrepancy between our results and previous studies may be due to differences in sample size, target populations, or the timing of conducting the studies.

Another reason was described in previous studies: younger students tended to be more worried about their future education and the ability to pay for college education than older students, and therefore spent more time on social media than older people did during the pandemic. The high frequency of news related to the COVID-19 pandemic, exposed younger students “always active” to a greater amount of input, increasing the risk of developing anxiety and worsening their mental health [57,58].

During the e-learning period, students felt that physical or mental health was not good, reporting 14 unhealthy days on average during the last month before the interview.

A total of 47% of them reported more than 14 unhealthy days (frequent unhealthy days) in the past 30 days. These findings were very worrying when compared to the mean of unhealthy days (3.0) collected in 2017–2020 in Italy in adults aged 18–34 years [59], or when comparing the percentage of frequent unhealthy days found in a prepandemic study of online university students (15.1%) [60]. Nevertheless, the unhealthy index was not related to e-learning satisfaction in our study.

The stress perceived was moderate in our sample (mean score = 19), but higher stress scores were related to lower satisfaction scores, it was, in fact, the only factor inversely associated with e-learning satisfaction (beta= −0.121; *p* < 0.001). Before the COVID-19 pandemic, some studies had already shown that stress in students could influence academic performance: students with high levels of stress had a worse academic performance [61,62]. Another study showed that the quality of stress influenced academic performance: those who experienced unfavourable stress had a worse performance [63].

Some studies reported a psychological impact of COVID-19 on students [64,65]. The association between stress and e-learning satisfaction found in our study was in line with the recent literature. In a report published during the COVID-19 pandemic, an increased need for psychological counseling of students was evidenced due to marked stress levels as a result of the drastic change in the learning environment [66].

Our data emphasized that e-learning satisfaction was strongly positively related to the social presence score, as confirmed by previous research [67,68]. This result was obvious and completely in line with the concept of social support as a positive and adaptive coping strategy in stressful conditions, such as in the COVID-19 pandemic.

D. R. Grafin [69], in line with what we reported, stated that during a period of forced distancing, such as that caused by the COVID-19 pandemic, online connections, e-learning, and therefore virtual classrooms allowed students to stay in touch with each other and continue their educational activities using digitized material and courses. Online platforms, therefore, provide a solution that allows people to maintain continuity in activities undertaken before the pandemic through live-streaming services while also providing the opportunity to create new connections and interests.

Another goal of our study was related to the investigation of the coping strategies among college students during COVID-19 e-learning. Recent research showed that a positive coping style could help students deal with problems in a more rational way, and could contribute to stress reduction [70].

During the COVID-19 pandemic, more negative coping strategies and fewer active coping styles were related to higher psychological stress [71,72].

A survey of a sample of Chinese medical students showed that more resilient individuals, who were therefore more adaptive to stressful situations, were more likely to use positive coping styles [45]. The same study stated that positive coping was beneficial to medical students in coping positively with Web-based lectures [70].

Our explorative univariate analysis of our study showed that e-learning satisfaction was significantly positively related with “Active coping”, “Positive reframing”, “Planning”, and “Religion”, while students who had behavioral disengagement or substance use as coping strategies were less satisfied.

The multivariable model, controlling for all factors, confirmed that “Religion” was positively related to e-learning satisfaction (beta = 0.303; *p* < 0.05).

Religion, especially in stressful periods., is an important coping strategy for dealing with particularly stressful situations; in troubled times it offers hope, perspective, and meaning, providing greater protection to those predisposed to mental health problems [73,74]. Another finding of the study was that e-learning satisfaction was associated with the coping strategy of “Self-Blame” (beta = 0.435; *p* < 0.05). Our results were supported by a recent study showing that one of the main coping strategies used in Moroccan students was a predictor of psychological well-being [75]. Self-blame as a coping strategy determines the attribution of causal responsibility and/or intentionality to oneself with respect to the outcome of an event. Unpredictable and uncontrollable external stress factors may lead to self-blame and subsequent internalization of stable negative traits [76,77].

The positive relationship between e-learning and “Self-Blame” coping was surprising, and it could be explained by taking into account that, as reported by Elin Thuen and Edvin, “Self-Blame” is a coping strategy related to academic or scholarly problems [78].

We plan to engage in other studies to investigate this aspect, because students with academic problems may prefer distance learning due to the lack of real interaction, which is replaced with virtual interaction, with fewer opportunities to compare themselves to others. This result also should be investigated with respect to the attitudes toward online learning, as well as with respect to the economic difficulties related to university life.

The present study had some limitations. First, it was based on a convenience sample of students from a single university; therefore, the lack of random selection, as well as the possible selection bias related to recruitment, limited the generalizability of the results [40]. Other limitations were related to the self-report measures, as well as the fact that the study design did not permit conclusions regarding causality.

Although all measures were obtained during the COVID-19 pandemic, none included specific pandemic-related factors or the technological skills of the participants or teachers, and there was a lack of previous psychological evaluation of the participants and their coping strategies, as well as other social variables.

## 5. Conclusions

Despite some limitations, the study indicated some implications for the future when considering extraordinary events that could repeat themselves and upset academic life.

The study was conducted after one year of virtual learning usage due to the pandemic, so the students were not living in strict quarantine, and they restarted partial socialization. Despite the extemporized change from traditional to distance learning, the students revealed a positive propensity to use e-learning, especially medical students and older students, but not stressed students.

Evidence showed that the use of software could support teachers and children with psychological distress or disabilities [79]. It is possible that virtual learning by medical students, who generally report higher levels of stress, has advantages such as saving travel time, cost reduction, and improved time organization. Furthermore, various studies carried out before the pandemic showed a high prevalence of stress in medical students [80,81]. Perhaps the additional stress may have had less of an effect on them during the pandemic because they already showed a high level of stress, which may explain their higher e-learning satisfaction.

The positive relationships found between e-learning satisfaction and religious coping confirmed that during a disaster, the most frequent coping strategy is religious coping. The association between e-learning satisfaction and self-blame coping was very interesting and should be investigated, as the literature does not offer many possibilities of interpretation.

In conclusion, the relationship between coping strategies and satisfaction with e-learning represents the need to provide dual training in the educational context.

The university should also be an interactive community in virtual learning; our results suggested a reflection on how we can ensure, in addition to training, the maturation of specific coping strategies, tools for better stress management, and greater adaptability not only in an emergency environment, ensuring interpersonal growth and greater individual gratification.

## Figures and Tables

**Table 1:** Student characteristics.

Characteristics	*N* = 471 *N* (%) or Mean (SD)
Gender (Male)	136 (29%)
Age	25 (5.7)
Residence	
Southern Italy	295 (63%)
Central Italy	139 (29%)
Northern Italy	36 (8%)
Attending course	
Medicine	223 (47%)
Health science	131 (28%)
Others	117 (25%)

**Table 2:** E-learning Satisfaction Scale.

Items	Mean	SD	MIN	Max
I was able to learn from the online lessons	3.777	1.057	1	5
I was stimulated to do additional reading or research on topics discussed	2.998	1.261	1	5
I would like to participate in another online course in the future	2.19 9	1.514	1	5
I am satisfied with the technology used in this course	3.512	1.775	1	5
The diversity of topics in the online course prompted me to participate in the discussions	2.643	1.288	1	5
I had difficulty learning how to use the platform	1.747	1.067	1	5
I enjoyed participating in the course	3.172	1.415	1	5
Professor was available and helpful in facilitating the use of the platform	3.592	1.446	1	5
I had difficulty concentrating	3.382	1.395	1	5
Online lessons facilitated the learning of the topics of the course	2.834	1.242	1	5

**Table 3:** Stress, health-related quality of life, sociality, and coping strategies.

	Mean (SD)
Stress (GHQ-12)	19.4 (7.0)
Social Presence (SPRES)	36 (8.7)
Health-related Quality of Life (HRQoL: Summary Index of Unhealthy Days)	14 (10.0)
Brief Cope Scale	
Self-distraction	5.3 (1.9)
Active coping	6.2 (1.3)
Denial	3.1 (1.4)
Substance use	2.5 (1.3)
Use of emotional support	5.1 (1.7)
Use of instrumental support	5.3 (1.7)
Behavioral disengagement	3.3 (1.4)
Venting	4.9 (1.5)
Positive reframing	5.5 (1.5)
Planning	6.4 (1.3)
Humor	4.2 (1.5)
Acceptance	6.0 (1.3)
Religion	3.2 (1.7)
Self-blame	5.9 (1.4)

**Table 4:** Correlation analysis.

	E-Learning Satisfaction	
Variables	Spearman′s rho	*p*-Value
Age	0.3023	0.0000
Stress (GHQ-12)	−0.4350	0.0000
Social Presence (SPRES)	0.7911	0.0000
Summary Index of Unhealthy Days (HRQoL)	−0.1830	0.0001
Brief Cope Scale		
Self-distraction	0.0304	0.5111
Active coping	0.1664	0.0003
Denial	0.0018	0.9694
Substance use	−0.1471	0.0014
Use of emotional support	0.0038	0.9347
Use of instrumental support	0.0430	0.3521
Behavioral disengagement	−0.1092	0.0178
Venting	0.0417	0.3668
Positive reframing	0.1309	0.0044
Planning	0.0927	0.0443
Humor	0.0070	0.8796
Acceptance	0.0798	0.0837
Religion	0.2853	0.0000
Self-blame	−0.0348	0.4518

**Table 5:** Factors associated with e-learning satisfaction (*n* = 471).

	Model I			Model II			Model III		
Variables	Beta	95%CI		Beta	95%CI		Beta	95%CI	
Gender (Ref: female)	−0.315	−1.687	1.056	−1.072 *	−1.930	−0.213	−0.755	−1.650	0.140
Age	0.384 **	0.273	0.495	0.124 **	0.534	0.195	0.123 **	0.051	0.194
Attending course (Ref: Others)									
Medicine	1.576 *	0.005	3.147	1.224 *	0.258	2.190	1.120 *	0.232	2.171
Health science	1.851 *	0.124	3578	0.287	−0.780	1.355	0.136	−0.935	1.206
Residence (Ref: Southern Italy)									
Central Italy	0.481	−0.905	1.868	0.711	−0.780	1.569	0.390	−0.482	1.262
Northern Italy	−0.0131	−2.445	2.419	0.663	−0.845	0.172	0.830	−0.680	2.341
Stress (GHQ-12)				−0.091 *	−0.159	−0.022	−0.121 **	−0.193	−0.050
Health-related Quality of Life (HRQoL: Summary Index of Unhealthy Days)				0.022	−0.021	0.066	0.013	−0.030	0.057
Social Presence (SPRES)				0.614 **	0.562	0.665	0.601 **	0.549	0.653
Brief Cope Scale									
Self-distraction							0.119	−0.144	0.382
Active coping							−0.224	−0.635	0.186
Denial							−0.158	−0.489	0.172
Substance use							−0.065	−0.392	0.261
Use of emotional support							−0.253	−0.604	0.098
Use of instrumental support							0.332	−0.009	0.672
Behavioral disengagement							0.086	−0.267	0.440
Venting							0.124	−0.170	0.439
Positive reframing							0.189	−0.110	0.487
Planning							−0.222	−0.625	0.181
Humor							−0.169	−0.460	0.122
Acceptance							−0.103	−0.429	0.223
Religion							0.303 *	0.604	0.545
Self-blame							0.435 *	0.135	0.735
R2	0.112			0.667			0.686		
F	9.746			102.431			42.451		
∆R2	0.112			0.555			0.019		
∆F	9.746			255.638			1.963		

Note: ∆F, change in F; ∆R2, change in variance; Beta, regression coefficient; F, F statistic from model ANOVA; R2, variance explained by the independent variables; Health-related Quality of Life: Summary Index of Unhealthy Days; Sociality: Social Presence (SPRES), * *p* < 0.05. ** *p* ≤ 0.001.

## Data Availability

The data analyzed in this study are not available to outside researchers due to privacy issues.

## Supplementary Materials

The following supporting information can be downloaded at: https://www.mdpi.com/article/10.3390/ijerph19138214/s1. The questionnaire used in the study (translated in English) is provided.
