# Supplementary material for: E-Learning Satisfaction, Stress, Quality of Life, and Coping: A Cross-Sectional Study in Italian University Students a Year after the COVID-19 Pandemic Began
Source: Int J Environ Res Public Health. 2022 Jul 5;19(13):8214. doi: 10.3390/ijerph19138214 (PMC9266753; doi:10.3390/ijerph19138214)
Supplement: Supplementary file 1 [file ijerph-19-08214-s001.zip › ijerph-1752885-supplementary.pdf]

E-learning satisfaction questionnaire

Healthy Days Core Module (CDC HRQOL– 4)

1. Would you say that in general your health is

Poor – Fair – Good - Very good - Excellent

2. Now thinking about your physical health, which includes physical illness and injury, for how many days during the past 30 days was your physical health not good?
_____________________ (Number of days)

3. Now thinking about your mental health, which includes stress, depression, and problems with emotions, for how many days during the past 30 days was your mental health not good?
___________________ (Number of days)

4. During the past 30 days, for about how many days did poor physical or mental health keep you from doing your usual activities, such as self-care, work, or recreation?
___________________ (Number of days)

Satisfaction Scale

l=Strongly Disagree, 2=Disagree, 3=Uncertain, 4=Agree, 5=Strongly Agree

1. I was able to learn through the medium of CMC.
2. I was able to learn from GlobalEd discussions.
3. I was stimulated to do additional reading or research on topics discussed in GlobalEd.
4. I learned to value other points of view.
5. As a result of my experience with GlobalEd, I would like to participate in another computer conference in the future.
6. GlobalEd was a useful learning experience.
7. Projects like GlobalEd enhance face-to-face on-campus courses.
8. As a result of my participation in GlobalEd, I made acquaintances electronically in other parts of the country/world.
9. The diversity of topics on GlobalEd prompted me to participate in the discussions.
10. I put in a great deal of effort to learn the CMC system to participate in GlobalEd.

Social Presence Scale

Likert scale used: l=Strongly Disagree, 2=Disagree, 3=Uncertain, 4=Agree, 5=Strongly Agree

1. Messages on GlobalEd were impersonal.
2. CMC is an excellent medium for social interaction.
3. I felt comfortable conversing through this textbased medium.

4. I felt comfortable introducing myself on GlobalEd.
5. The introductions enabled me to form a sense of online community.
6. I felt comfortable participating in GlobalEd discussions.
7. The moderators created a feeling of an online community.
8. The moderators facilitated discussions in the GlobalEd conference.
9. Discussions using the medium of CMC tend to be more impersonal than face-to-face discussions.
10. CMC discussions are more impersonal than audio teleconference discussions.
11. CMC discussions are more impersonal than video teleconference discussions.
12. I felt comfortable interacting with other participants in the conference.

Short General Health Questionnaire (GHQ 12)

Have you recently?

1. Been able to concentrate on what you're doing?

   Better than usual - Same as usual - Less than usual - Much less than usual
2. Lost much sleep over worry?

   Not at all - No more than usual - Rather more than usual - Much more than usual
3. Felt you were playing a useful part in things?

   More so than usual - Same as usual - Less useful than usual - Much less useful
4. Felt capable of making decisions about things?

   More so than usual - Same as usual - Less so than usual - Much less capable
5. Felt constantly under strain?

   Not at all - No more than usual- Rather more than usual - Much more than usual
6. Felt you couldn't overcome your difficulties?

   Not at all - No more than usual - Rather more than usual - Much more than usual
7. Been able to enjoy your normal day-to-day activities?

   More so than usual - Same as usual - Less so than usual - Much less than usual
8. Been able to face up to your problems?

   More so than usual - Same as usual - Less so than usual - Much less able
9. Been feeling unhappy and depressed?

   Not at all - No more than usual - Rather more than usual - Much more than usual
10. Been losing confidence in yourself?

    Not at all - No more than usual - Rather more than usual - Much more than usual
11. Been thinking of yourself as a worthless person?

    Not at all - No more than usual - Rather more than usual - Much more than usual
12. Been feeling reasonably happy, all things considered?

More so than usual - About same as usual - Less so than usual - Much less than usual

Brief-COPE (Brief-COPE)

Instructions: The following questions ask how you have sought to cope with a hardship in your life. Read the statements and indicate how much you have been using each coping style.

(I haven't been doing this at all - A little bit - A medium amount - I've been doing this a lot)

1) I've been turning to work or other activities to take my mind off things.
2) I've been concentrating my efforts on doing something about the situation I'm in.
3) I've been saying to myself "this isn't real".
4) I've been using alcohol or other drugs to make myself feel better.
5) I've been getting emotional support from others.
6) I've been giving up trying to deal with it.
7) I've been taking action to try to make the situation better.
8) I've been refusing to believe that it has happened.
9) I've been saying things to let my unpleasant feelings escape.
10) I've been getting help and advice from other people.
11) I've been using alcohol or other drugs to help me get through it.
12) I've been trying to see it in a different light, to make it seem more positive.
13) I've been criticizing myself.
14) I've been trying to come up with a strategy about what to do.
15) I've been getting comfort and understanding from someone.
16) I've been giving up the attempt to cope.
17) I've been looking for something good in what is happening.
18) I've been making jokes about it.
19) I've been doing something to think about it less, such as going to movies, watching TV, reading, daydreaming, sleeping, or shopping.
20) I've been accepting the reality of the fact that it has happened.
21) I've been expressing my negative feelings.
22) I've been trying to find comfort in my religion or spiritual beliefs.
23) I've been trying to get advice or help from other people about what.
24) I've been learning to live with it.
25) I've been thinking hard about what steps to take.
26) I've been blaming myself for things that happened.
27) I've been praying or meditating.
28) I've been making fun of the situation.
